# Multiturn Large Language Model–Based Conversational Agents for Patients With Cancer and Caregivers: Scoping Review

**DOI:** 10.2196/96241

**Published:** 2026-07-21

**Authors:** Yeongrok Jeong, Hyejeon Cha, Eunyoung E Suh

**Affiliations:** 1 Center for World-leading Human-care Nurse Leaders for the Future by Brain Korea 21 (BK21) Four Project, College of Nursing, Research Institute of Nursing Science, Seoul National University, 103 Daehak-ro, Jongno-gu, Seoul, 03080, Republic of Korea, 82 027408807; 2 College of Nursing, Seoul National University, Seoul, Republic of Korea

**Keywords:** chatbot, large language model, LLM chatbot, conversational agent, artificial intelligence, generative artificial intelligence, generative AI chatbot, cancer, neoplasms

## Abstract

**Background:**

Large language model (LLM)–based conversational agents are increasingly used in health care, yet their capacity to support genuine multiturn dialogue remains underexplored. In oncology, where patients and caregivers experience complex informational and emotional needs throughout the disease trajectory, conversational agents may support information provision, symptom consultation, and emotional assistance. However, research specifically examining multiturn conversational agents designed for patients with cancer and informal caregivers remains limited.

**Objective:**

This scoping review aimed to map the research landscape of LLM-based multiturn conversational chatbots developed for patients with cancer and informal caregivers, focusing on system design, intervention purposes, evaluation approaches, safety considerations, and transparency of LLM-related components.

**Methods:**

This scoping review followed the Joanna Briggs Institute methodology and PRISMA-ScR (Preferred Reporting Items for Systematic Reviews and Meta-Analyses Extension for Scoping Reviews) guidelines. Six databases—PubMed, Embase, Scopus, Web of Science, CINAHL, and PsycINFO—were searched for studies published between January 2022 and January 2026, with supplementary searches conducted in IEEE Xplore Digital Library and ACM Digital Library in May 2026. Studies were included if they described LLM-based chatbots designed for patients with cancer or informal caregivers that supported multiturn conversational interaction. Two reviewers independently conducted the study selection and data extraction.

**Results:**

Eight studies met the inclusion criteria. Most studies focused on prototype development, with limited research evaluating clinical outcomes. ChatGPT-based models were the most commonly used LLMs, and retrieval-augmented generation techniques were applied in several studies. Chatbots were primarily designed for emotional support or information provision. Evaluation approaches varied widely, including response quality, psychological outcomes, and user experience. However, no studies evaluated interaction-level characteristics such as conversational continuity or context retention, and only 2 studies reported any conversational memory mechanism. Reporting on safety risks, mitigation strategies, prompt design, model parameters, and adherence to LLM reporting guidelines was often limited or absent.

**Conclusions:**

This scoping review identified only 8 studies on LLM-based multiturn conversational chatbots for patients with cancer and informal caregivers. The field remains at an early stage, characterized by prototype-oriented development, heterogeneous design and evaluation approaches, and inconsistent safety and transparency reporting. Future development should prioritize genuine conversational capability, safety management, and transparent reporting.

## Introduction

Large language models (LLMs) represent an advanced subset of natural language processing (NLP), capable of generating contextually appropriate responses through probabilistic text generation [1]. Chatbots using LLMs can generate responses in multiturn interactions while maintaining context, without relying on predefined rules or response scenarios [2,3]. Such capability distinguishes them from traditional rule-based NLP-based chatbots, revealing fundamental differences in design principles and conversational approaches [4,5]. This generative nature, however, introduces novel risk structures that are more pronounced than those in rule-based NLP systems [6,7].

Following the proliferation of LLMs, LLM-based conversational agents for diverse purposes—such as patient education, symptom consultation, emotional support, and treatment decision-making assistance—are rapidly emerging in the medical field, with accumulating evaluation studies [8]. In mental health and chronic disease management, LLM-based conversational agents have increasingly been developed to improve patient outcomes, demonstrating clinical benefits across conditions including depression, anxiety, eating disorders, and diabetes [9-11].

In oncology, where information needs and uncertainty are high during diagnosis and treatment, the potential application of LLM-based conversational agents is gaining attention [12,13]. For patients with cancer and informal caregivers, whose communication, informational, and emotional needs evolve throughout the disease course, multiturn interactions are a critical characteristic necessitating treatment as an independent analytical category [14,15]. Chatbot studies applying diverse LLMs, such as ChatGPT, are rapidly proliferating in this context, alongside advances in development frameworks including retrieval-augmented generation (RAG), knowledge graphs, and LangChain. However, existing literature tends to focus on performance comparisons at the single-turn question-answer level, making it difficult to distinguish and understand the design and evaluation characteristics specific to patient-directed conversational agents [16].

Multiturn conversational interaction refers to systems that maintain context by integrating the current message and previous conversation history when generating responses [17,18]. Yet, in LLM-based conversational agents for patients with cancer or informal caregivers, multiturn capability is rarely evaluated or explicitly implemented in practice, despite being considered inherent to conversational agents.

Simultaneously, LLMs carry safety risks, including hallucinations and responses that may pose psychological risks to vulnerable patients [6,7,19]. Although expert oversight and escalation systems have been suggested as mitigation strategies, systematic reporting of safety risks and mitigation approaches in LLM-based oncology chatbot studies remains limited.

Furthermore, if reporting on LLM-specific components—such as prompts, model settings, and external knowledge integration methods—is insufficient, reproducibility and interpretability may be limited [20]. Reporting guidelines, such as TRIPOD-LLM (Transparent Reporting of a Multivariable Prediction Model for Individual Prognosis or Diagnosis–Large Language Models) and CHART (Chatbot Assessment Reporting Tool), have been developed to address this concern [21,22]; however, the extent to which LLM-based chatbot studies in oncology adhere to these guidelines remains unclear.

Several reviews have examined related topics in oncology. Wang et al [23] focused primarily on rule-based systems predating the LLM era. Chen et al [24] evaluated the medical accuracy of LLM-based chatbots in oncology across diagnostic and management tasks, but centered on single-turn performance rather than patient-facing conversational interaction. Jiang et al [25] synthesized conversational agent interventions in cancer care without restricting to LLM-based systems, identifying LLMs as a key emerging technology to enhance personalization and sustained engagement. To our knowledge, none of these reviews specifically examined the unique characteristics of LLM-based chatbots such as system design, safety, and transparency in the context of multiturn conversational interaction with patients with cancer and informal caregivers.

Therefore, this scoping literature review aims to identify the overall research landscape of LLM-based conversational chatbot studies developed for patients with cancer and their caregivers and sets the following research questions:

1. What types of LLM-based conversational chatbots have been developed for patients with cancer and caregivers, and what are their key design elements?
2. What are the primary intervention goals of the developed LLM-based chatbots?
3. How and at what level were LLM-based chatbots evaluated?
4. What safety risks were reported for LLM-based chatbots, and what mitigation strategies were used?
5. To what extent were the key LLM-related components of the chatbot development process transparently reported to support reproducibility?

## Methods

This scoping systematic review was conducted according to the Joanna Briggs Institute (JBI) scoping systematic review methodology [26] and reported according to the PRISMA-ScR (Preferred Reporting Items for Systematic Reviews and Meta-Analyses Extension for Scoping Reviews) guidelines (Checklist 1) [27]. The protocol for this study was preregistered in the Open Science Framework (OSF) [28], and a critical appraisal of individual studies was not performed.

### Search Strategy

#### Search Sources

A pilot search for relevant literature was conducted in 3 databases—PubMed, Embase, and Scopus—from January 20, 2026, to January 31, 2026. Based on the pilot search results, the search strategy and inclusion or exclusion criteria were refined. An expanded search was then conducted from February 9, 2026, to February 13, 2026, across 6 databases initially: PubMed, Embase, Scopus, Web of Science, CINAHL, and PsycINFO. IEEE Xplore Digital Library and ACM Digital Library were subsequently searched on May 10, 2026, to ensure comprehensive coverage of computer science and AI literature.

#### Search Terms

Search terms were designed to comprehensively identify studies on LLM-based interactive chatbots targeting patients with cancer and their informal caregivers. The search query comprised four blocks: (1) cancer, (2) conversational agent or chatbot, (3) LLM or generative AI, and (4) patient or informal caregiver. Each block combined controlled vocabulary (eg, MeSH/EMTREE) with free-text terms using AND operators. The same search structure was applied across all databases; the full search terms for each database are presented in Multimedia Appendix 1.

### Study Eligibility Criteria

This review included peer-reviewed journal articles and conference proceedings, encompassing various research designs such as experimental studies, observational studies, mixed methods studies, system development and evaluation studies, and review studies. Conversely, literature lacking systematic research design or results reporting, such as editorials, opinion pieces, and conference abstracts, was excluded. The literature was limited to English-language articles published between January 2022 and January 2026.

### Participants

The participants for this scoping review were defined as studies addressing LLM-based chatbots developed primarily for patients with cancer and their informal caregivers, including family members, spouses, and other unpaid supporters involved in the care of patients with cancer. Participants were restricted to adult patients with cancer and adult caregivers. Studies including pediatric participants were included in this review if adult patients were also a primary focus.

### Concept

The core concept of this review was interactive chatbots developed using LLMs. Multiturn conversational interaction was operationally defined as systems designed to support iterative dialogue, including those that (1) explicitly generate responses based on previous conversational context, (2) are architecturally configured to enable context-maintaining exchanges, or (3) are developed with the explicit intent of facilitating ongoing conversational interaction with users. Conversely, conversational systems implemented solely using rule-based NLP techniques or predefined response scenarios were excluded from this study’s scope. Additionally, single-turn chatbots evaluated solely for accuracy, readability, and so forth, without presupposing interaction, were excluded.

### Context

The purpose of this review is to identify the overall research landscape of LLM-based conversational chatbot studies developed for patients with cancer and their caregivers. Therefore, as it systematically organizes the purpose, design approach, application environment, and evaluation methods of the technology, it includes studies conducted in various contexts such as hospitals, homes, and online.

### Study Selection

This systematic review identified relevant studies from databases, removed duplicates, performed an initial screening based on titles and abstracts, and reviewed full texts to determine final inclusion according to the predefined inclusion and exclusion criteria. Two researchers (YJ, HC) independently performed the literature screening and full-text review. Disagreements in selection were resolved through discussion and consensus. To ensure efficiency and transparency in the selection process, the web-based systematic review program Rayyan (Rayyan Systems Inc.) was used. The selection process and results were reported using the PRISMA-ScR flowchart.

Some included studies were identified as borderline cases. Given the limited number of studies explicitly addressing multiturn LLM-based conversational agents in oncology, an inclusive approach was adopted to comprehensively capture the emerging research landscape in this field. The initial judgments of each author and the consensus process for all included studies are documented in Multimedia Appendix 2.

### Data Extraction and Data Synthesis

Data extraction was performed independently by 2 researchers using a predefined data extraction form, which was refined after pilot testing on a subset of studies. Data extraction was recorded using an electronic database tool (Notion), with a third researcher (ES) mediating when necessary.

Data extraction items included the following: (1) authors and publication year, (2) study country and setting, (3) study design and type, (4) participant characteristics, (5) primary purpose of the chatbot, (6) type of LLM used and system configuration, (7) evaluation design and key evaluation results, (8) reporting on safety and risk management, and (9) reporting on transparency or reproducibility elements related to the LLM.

The level of reporting on transparency and reproducibility of the LLM-related components was classified according to predefined criteria, adapted from reporting elements specified in TRIPOD-LLM [21]. The level of prompt reporting was assessed based on whether (1) system prompts, (2) user prompts, and (3) specific descriptions of model settings or parameters were provided. Complete reporting was defined as cases where all 3 elements were explicitly described, while partial reporting was defined as cases where only some elements were reported. Cases in which relevant information was not explicitly described were classified as not reported.

RAG was classified as applied when the term was explicitly used in the study or when the study described integrating information from external documents prior to response generation. Additionally, safety risk factors were categorized based on the risk factors described in each study.

The extracted data were organized into tables and diagrams according to study characteristics and key concepts, and the results were presented using descriptive mapping.

## Results

### Search Results

**Figure 1.**
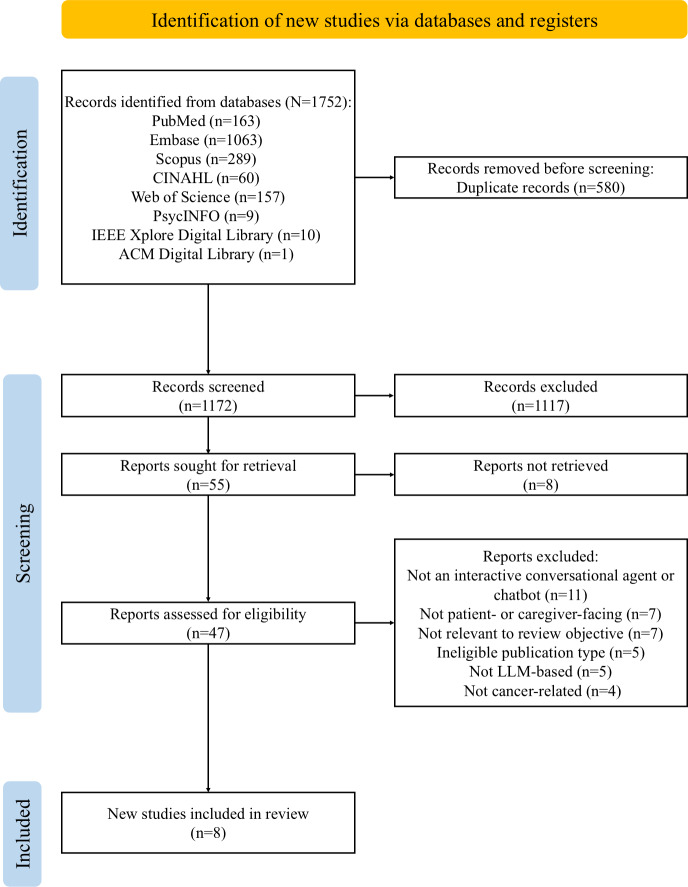
PRISMA (Preferred Reporting Items for Systematic Reviews and Meta-Analyses) flow diagram showing the study selection process.

This review searched a total of 8 databases according to a predefined search protocol. The search and selection process is presented in Figure 1. The initial search identified a total of 1752 documents, including books, conference proceedings, and journal articles. After removing 580 duplicate documents, 1172 documents were included for title and abstract screening.

Following the title and abstract screening, a total of 1117 documents were excluded, and 55 were selected for full-text review. Excluded documents were categorized based on predefined inclusion and exclusion criteria, with some documents meeting multiple exclusion criteria simultaneously. The specific distribution of exclusion reasons at the title and abstract stage is presented in Multimedia Appendix 3.

During the full-text review stage, 47 of the 55 articles were reviewed, excluding 8 articles with restricted full-text access. These comprised 1 oral abstract, 3 conference abstracts, 2 ePosters, 1 book chapter, and 1 news or views article, none of which met our inclusion criteria. Articles excluded during the full-text review were categorized based on 1 primary exclusion reason. The main exclusion reasons were as follows: not being an interactive chatbot (11 studies; eg, studies generating tailored health education materials using LLMs without conversational interaction, or evaluating the accuracy of single-turn question-answering systems), not primarily targeting patients or caregivers (7 studies), not addressing a cancer-related context (4 studies), not using an LLM (5 studies), publication type not meeting inclusion criteria (5 studies), and insufficient relevance to the study objectives (7 studies).

Ultimately, a total of 8 studies were included in this scoping review. The limited number of included studies reflects that research applying LLM-based interactive chatbots to patients with cancer and caregivers is a relatively recent field of study.

### General Description of the Studies

A total of 8 studies were included in this systematic review (Table 1). Two studies were reported in 2024 and 6 in 2025. Countries where studies were conducted included Germany, Japan, South Korea, the United States, Turkey, India, China, and the United Kingdom, with 1 study each from these countries. Publication types comprised 5 journal articles and 3 conference proceedings.

Regarding study design, development studies were the most common (5), followed by clinical evaluation studies (2) and usability evaluation studies (1). Seven studies developed chatbots exclusively for patients with cancer, while 1 study developed a chatbot exclusively for informal caregivers. No included study developed a chatbot for joint use by both patients and caregivers. Three studies specified breast cancer as the cancer type, while the remaining 5 studies did not restrict to a specific cancer type.

Two studies targeted newly diagnosed patients with cancer within 1 year of diagnosis, while 6 studies did not restrict by disease stage or timing of diagnosis. One study included a control group, 2 applied a pre-post design, and 5 did not use a comparison group, indicating varied comparative designs across studies. Overall, the included studies showed heterogeneous characteristics in terms of research design, participant characteristics, and comparison methods.

**Table 1.** General characteristics of the included studies.

Characteristics	Number of studies
Year of publication	
2024	2 [29,30]
2025	6 [31-36]
Country	
Germany	1 [33]
Japan	1 [34]
Republic of Korea	1 [30]
The United States	1 [36]
Turkey	1 [31]
India	1 [32]
China	1 [29]
The United Kingdom	1 [35]
Type of publication	
Journal article	5 [29-31,33,34]
Conference paper	3 [32,35,36]
Study design	
Clinical evaluation	2 [31,34]
Development study	5 [29,30,32,33,35]
Usability study	1 [36]
Target population	
Patients	7 [29-35]
Caregivers	1 [36]
Cancer type	
Breast cancer	3 [29,33,35]
Not restricted	5 [30-32,34,36]
Disease status	
Newly diagnosed (≤1 year since diagnosis)	2 [31,34]
Not restricted	6 [29,30,32,33,35,36]
Comparator design	
Controlled comparative design	1 [31]
Pre-post design	2 [34,36]
No comparator applied	5 [29,30,32,33,35]

### Types and Design Characteristics of LLM-Based Chatbots

Analysis of the types of LLMs used in the studies showed that ChatGPT 4.0 accounted for the largest share with 5 studies. ChatGPT 3.5, ChatGPT 3.5 Turbo, and Gemini were each used in 1 study. Additionally, some studies used various LLMs such as Gemini, Llama 3.1, Mistral 7B, and PHI 3.5 to perform model comparisons (Table 2).

Five studies applied RAG techniques, while 3 studies did not report on RAG application. Regarding knowledge sources used by chatbots, clinical practice guidelines were the most common source, used in 3 studies. Educational materials, books, and published literature were each used in 1 study. Conversely, 3 studies did not explicitly specify their knowledge sources.

Three studies explicitly used frameworks like LangChain or knowledge graphs for chatbot development, while the remaining 5 did not report on frameworks. Overall, the studies included in this literature review centered on ChatGPT-based models. However, heterogeneity was observed in model composition, with some studies using open-weight models for comparative evaluations. Differences were also noted in the level of reporting regarding knowledge sources and system implementation methods.

Meanwhile, the level of reporting on conversational memory handling was also limited. Only 1 study implemented embedding-based memory retrieval, and 1 reported history-based memory retention. Conversely, the remaining 6 studies did not present any method for maintaining context.

**Table 2.** Large language model (LLM)–specific design characteristics of included studies^a^^b^.

Characteristics	Number of studies
LLM profile (multiple models possible)	
ChatGPT 3.5	1 [30]
ChatGPT 3.5 Turbo	1 [29]
ChatGPT 4.0	5 [31-34,36]
Gemini	1 [32]
Llama 3.1	1 [35]
Mistral 7B	1 [35]
PHI 3.5	1 [35]
RAG^c^	
Applied	5 [30,32,33,35,36]
Not reported	3 [29,31,34]
Knowledge source	
Clinical guideline	3 [30,32,33]
Educational material	1 [33]
Published literature	1 [35]
Textbook-based sources	1 [30]
Not reported	3 [29,31,34]
Development framework	
LangChain	3 [30,32,36]
Knowledge graph	1 [32]
Not reported	5 [29,31,33-35]
Conversational memory handling	
Embedding-based memory retrieval	1 [29]
History-based memory retention	1 [34]
Not reported	6 [30-33,35,36]

a “Not reported” indicates that the item was not explicitly described in the study.

b Study [35] evaluated 3 large language models comparatively, and all models assessed are listed.

c RAG: retrieval-augmented generation.

### Primary Purposes of LLM-Based Chatbots

Analysis of the primary purposes of the developed chatbots revealed 4 categories: emotional support, information provision, enhancing user convenience, and analyzing user experience. Three studies focused on emotional support as their primary purpose, aiming to provide psychological support centered on reducing anxiety, depression, and stress. Three studies focused on information provision as their primary purpose, developing chatbots that provide personalized responses to cancer-related questions and deliver medical information.

Meanwhile, 1 study primarily aimed to support clinical research by automating the collection and organization of patient-reported outcomes (PRO) data. Another study primarily focused on exploring the interaction experience with conversational AI support agents, targeting caregivers as the main subjects.

Overall, the included studies used LLM-based chatbots as tools for information provision or emotional support. Some studies focused on enhancing clinical convenience or understanding user experience and interaction patterns.

### Evaluation Approaches and Outcome Domains

Analysis of evaluation methods and domains revealed that the assessment approaches and measured outcome domains varied significantly across studies (Table 3). One study evaluated participants’ psychological outcomes using validated measurement tools, while another reported psychological states using a predefined scoring scale. Additionally, 1 study focused its evaluation on user experience and perceptions.

Four studies evaluated the response performance of LLM-based chatbots, focusing on chatbot response accuracy, completeness, comprehension, consistency, and security or safety. One study focused on chatbot development and did not report separate evaluation results.

The studies included in this literature review were evaluated across diverse domains such as clinical outcomes, response quality, and user experience, revealing heterogeneous characteristics in the evaluation design and outcome domains. However, despite being multiturn interactive systems, no studies were identified that evaluated the continuity of conversation or the interaction process itself.

**Table 3.** Evaluation methods and outcome measures used in included studies.

Characteristics	Number of studies
General evaluation outcomes	
Psychological outcomes (validated instruments)	1 [31]
User-reported psychological states (nonvalidated measures)	1 [34]
User experience and perception	1 [36]
LLM^a^-specific evaluation outcomes	
Response accuracy	4 [29,31,33,35]
Response completeness	2 [29,33]
Response comprehension	3 [29,30,33]
Response consistency	1 [29]
Response security	2 [29,35]
No formal evaluation	1 [32]

a LLM: large language model.

### Reported Safety Risks and Mitigation Strategies

Analysis of safety risks and mitigation strategies reported in the included studies revealed that the level of safety-related reporting varied across studies (Table 4). Risks related to the reliability of chatbot responses were reported in 4 studies, while risks related to physical and psychological safety were identified in 1 study each. Conversely, 3 studies did not provide specific reports on safety risk factors.

Two studies specified expert intervention as a strategy to mitigate safety risks. Two studies applied an automated escalation system that provided emergency contacts to participants or connected them to external support systems during crisis situations. Additionally, 1 study applied a strategy to exclude models with low performance or potential harm. However, the remaining 3 studies did not report on risk mitigation strategies. While some studies proposed safety management strategies, systematic reporting of safety risk factors and mitigation strategies was limited.

**Table 4.** Reported safety risks and mitigation strategies in included studies.

Characteristics	Number of studies
Safety risks	
Response reliability	4 [29,31,34,35]
Psychological safety risk	1 [34]
Physical safety risk	1 [32]
Not reported	3 [30,33,36]
Mitigation strategies	
Expert involvement	2 [31,34]
Escalation systems	2 [32,34]
Removal of poorly performing models	1 [35]
Not reported	3 [30,33,36]

### Transparency and Reproducibility of LLM-Specific Components

Analysis of the reporting level for LLM-related components revealed that transparency in prompt design and system configuration varied across studies (Table 5). No study reported prompts in full, while 5 studies reported only partial components. Conversely, 3 studies did not report prompt design at all.

Only 1 study explicitly reported the system prompt as a prompt component, 2 reported the user prompt, and 2 reported model parameters. However, 3 studies did not report specific information about prompt components. Furthermore, only 1 study explicitly referenced LLM-based research reporting guidelines, while the remaining 7 studies made no mention of such guidelines.

**Figure 2.**
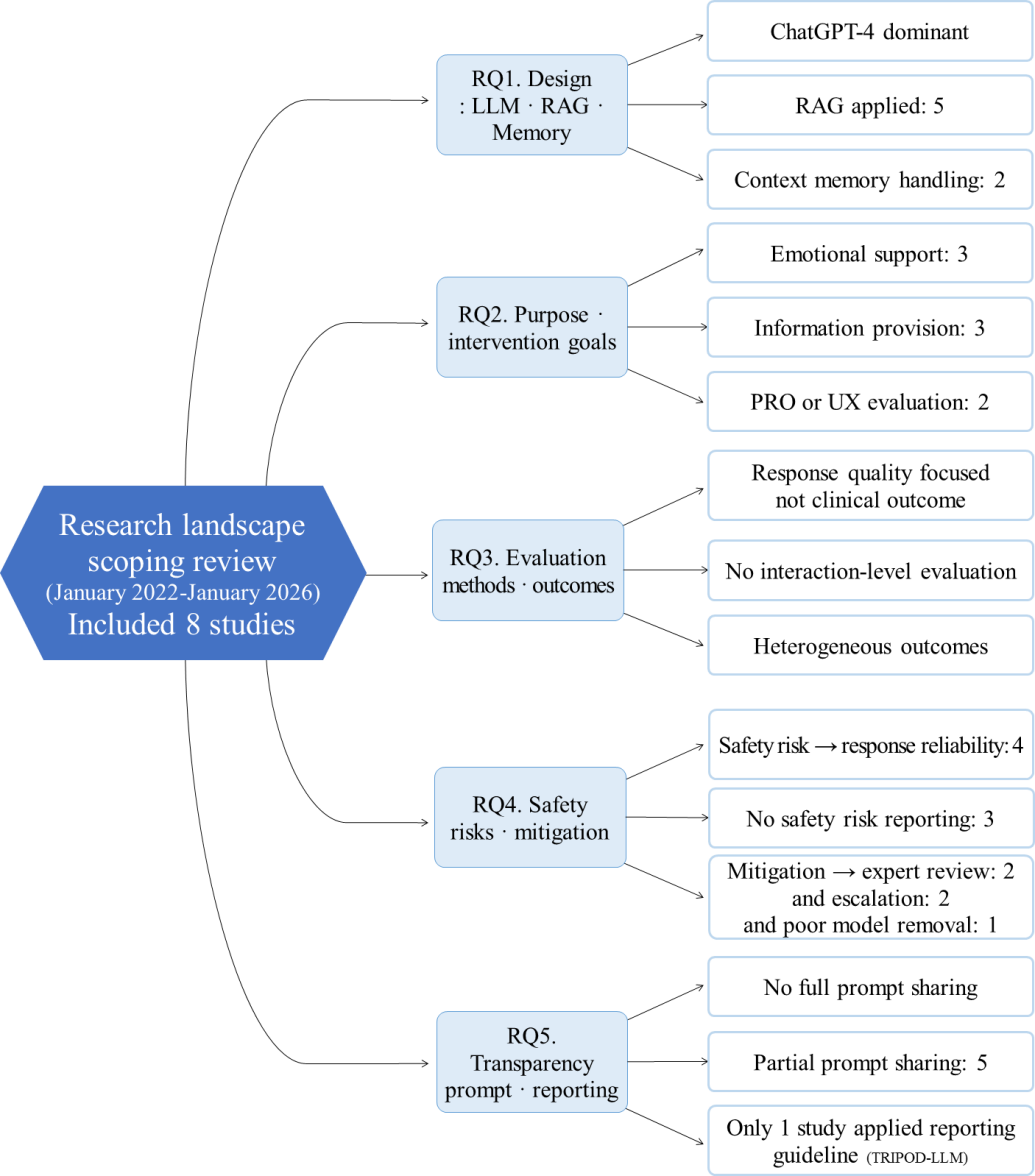
Conceptual map of the research landscape across 5 research questions (RQs). LLM: large language model; PRO: patient-reported outcome; RAG: retrieval-augmented generation; TRIPOD-LLM: Transparent Reporting of a Multivariable Prediction Model for Individual Prognosis or Diagnosis–Large Language Models; UX: user experience.

Overall, the included studies provided basic information on LLM selection and use, but reporting on reproducibility aspects, such as prompt design and system configuration, was limited. The research landscape across the 5 research questions is summarized in Figure 2.

**Table 5.** Transparency and reproducibility of large language model (LLM)–specific components.

Characteristics	Number of studies
Prompt reporting completeness	
Complete	0
Partial	5 [29,30,33-35]
Not reported	3 [31,32,36]
Prompt components reported	
System prompt	1 [33]
User prompt	2 [29,34]
Model parameters	2 [30,35]
Not reported	3 [31,32,36]
Reporting guideline adherence	
TRIPOD-LLM^a^	1 [33]
Not reported	7 [29-32,34-36]

a TRIPOD-LLM: Transparent Reporting of a Multivariable Prediction Model for Individual Prognosis or Diagnosis–Large Language Models.

## Discussion

### Principal Findings

The most significant finding of this review is the immaturity of the multiturn chatbot field in oncology. Multiturn interactions are often regarded as an inherent and fundamental feature of conversational agents. Nevertheless, studies that could be rigorously classified as genuinely multiturn were markedly limited. This scoping review identified 8 studies, of which only 2 explicitly reported any conversational memory mechanism. Most studies included in this review remained at the stage of system development and prototyping, with limited research applying comparative designs or validating clinical efficacy. While systematic assessments of patient-centered clinical outcomes were relatively scarce, most of the studies focused on response quality and safety verification.

In terms of design, ChatGPT-based models were primarily used, with some studies applying RAG techniques or external knowledge integration methods. The level of reporting on key design elements—such as knowledge sources, retrieval architecture, model configuration, and prompt composition—varied significantly across studies. Furthermore, reported safety risk factors, mitigation strategies, and transparency of LLM components were inconsistent across studies. Only 1 study explicitly referenced reporting guidelines.

Research on LLM-based oncology multiturn conversational chatbots is rapidly expanding but has not yet reached maturity. Key areas requiring further development include clarity of design architecture, validation of clinical efficacy, safety management strategies, and reporting standardization.

### Design and Intervention Characteristics of LLM-Based Oncology Chatbots

Overall, the included studies focused on prototype-level implementations based on commercial LLMs, exhibiting heterogeneity in model selection, knowledge integration strategies, and system design approaches. This design diversity can be interpreted as reflecting the exploratory application phase of the technology [37]. However, such diversity in chatbot design also complicates comparability between studies [20,38].

In oncology, LLM-based chatbots were often developed for emotional support and information provision. Some studies presented automation of collecting and organizing PRO data or exploring user experience and interaction patterns as primary objectives. This aligns with prior research indicating that LLM-based chatbots in oncology are applied for education and patient support [39,40].

Meanwhile, studies specifying a particular cancer type were primarily focused on breast cancer, and several studies targeted patients in the early stages of diagnosis. These patterns suggest that LLM-based chatbot interventions are initially being applied to patient groups with relatively standardized clinical guidelines and high informational and emotional support needs [13,41]. However, this bias toward specific cancer types and disease stages limits generalizability across oncology. Furthermore, despite being premised on multiturn interactions, studies explicitly addressing conversational memory handling were limited.

Most studies identified in this literature review focused on individual response-level performance metrics, such as chatbot response accuracy or completeness. No studies were found that evaluated the multiturn interaction process itself. Multiturn dialogue systems generate responses by considering previous utterances and conversation history. They are reported as a crucial technical element for providing sophisticated information and building psychological rapport by posing follow-up questions in real time [42,43]. Furthermore, the ability to generate consistent responses while maintaining long-term conversational context is presented as a major research challenge for LLM-based conversational systems [18]. Therefore, evaluating interaction-level factors, such as conversational continuity and context retention capability, may also be important considerations.

### Evaluation, Safety, and Transparency of LLM-Based Conversational Agents

Included studies tended to focus on response quality and safety verification, with limited comparative designs evaluating clinical efficacy. Furthermore, while some studies mentioned potential psychological risks, safety reporting was limited in a significant number of studies, and specific safety threat factors were not explicitly reported. Additionally, while evaluating the “quality” and “stability” of interactions is crucial for multisession interactive chatbots, reporting on this aspect was also limited.

LLMs are generative models based on probabilistic next-word prediction engines that can produce plausible-looking but factually inconsistent hallucinations [44,45]. Response accuracy varies across studies and models, but reported accuracy levels around 80% still imply the potential for generating inaccurate information [46-48]. Particularly, cancer treatment requires a multidisciplinary approach and complex decision-making. Therefore, the accuracy and reliability of information provided by LLM-based chatbots can significantly impact the decision-making and treatment processes of patients with cancer [24,49,50].

Mitigation strategies to reduce hallucinations and improve accuracy, such as RAG or knowledge graphs that connect LLMs with external evidence sources, have been proposed. At the clinical level, some studies proposed expert prereview as a safety measure [31,34] or introduced escalation mechanisms connecting to external support systems during crisis situations [34]. However, these mitigation strategies were not consistently applied or systematically evaluated across studies, and many studies did not sufficiently describe specific risk management strategies. Therefore, establishing a standardized framework to systematically define and report safety threats in LLM-based conversational chatbots is required.

With respect to transparency, reporting on LLM-related components was generally limited across the included studies, as noted in existing literature [20,51,52]. While some studies partially disclosed prompts, key elements such as system prompts, user prompts, and model parameters were often insufficiently described. Prompt engineering, the process of adjusting LLM responses through the input structure and instruction design, impacts response accuracy [53-56]. Furthermore, since LLMs respond sensitively to prompt structure without revealing their internal reasoning processes, prompt design is a critical variable in determining response appropriateness in medical settings [21,57-59]. Therefore, the failure to clearly report prompt structure and model settings may limit the interpretability and reproducibility of research findings [60].

Notably, only 1 included study explicitly referenced a reporting guideline—specifically, TRIPOD-LLM [21]—underscoring the limited adoption of standardized reporting frameworks in this emerging field. Standardized and transparent reporting is essential for building trust in the use of generative AI models in clinical practice [22]. Reporting guidelines such as TRIPOD-LLM, CHART, and CONSORT-AI have been developed to address this gap [21,22,61]. Future studies on LLM-based chatbots in oncology should explicitly adhere to these frameworks.

### Implications for Future Research and Practice

Future research should report model versions, knowledge update cycles, retrieval strategies, prompt systems, and system pipelines in standardized formats to enhance comparability and reproducibility across studies. For systems designed for multiturn interactions, it is necessary to describe conversational state management and context retention (conversational memory handling) strategies and apply interaction-level evaluation metrics such as dialogue consistency and safety. Furthermore, the applicability across diverse cancer types, disease stages, and user groups (patients and caregivers) should be systematically validated, and comparative designs enabling clinical efficacy assessment should be expanded. Finally, adherence to established reporting guidelines is strongly recommended to promote transparency and reproducibility in future studies.

### Limitations

This study has the following limitations. First, the search was restricted to English-language publications in 8 databases, so the possibility of omitting relevant studies cannot be ruled out. Second, due to the rapidly evolving nature of this research field, conference papers were included; however, some studies did not sufficiently report detailed methods and components, limiting interpretation. Third, consistent with the nature of a scoping review, methodological quality assessments of individual studies were not performed, preventing a systematic comparison and evaluation of the evidence levels across the included studies. Fourth, given the rapid pace of LLM research, studies published after the search cutoff (February 2026 for the primary databases and May 2026 for the supplementary AI or computer science databases) may not be captured in this review, and periodic updates are recommended to reflect the latest developments in this field.

### Conclusion

This scoping review systematically summarized the overall research landscape of LLM-based multiturn conversational chatbot studies developed for patients with cancer and their informal caregivers. Only 8 studies were included, most of which were recently reported prototype-focused development research, reflecting the emerging nature of this field. Heterogeneity among studies was found in design architecture, evaluation methods, safety management strategies, and the level of transparency. Notably, despite the conversational nature of the included systems, reporting on interaction-level evaluations, such as context retention strategies, was limited.

LLM-based oncology conversational chatbots show potential as promising tools for patients with cancer and caregivers. Genuine multiturn conversational capability should therefore be prioritized in future development. However, safety management and standardization of design and reporting must proceed concurrently. Future research should explicitly identify potential patient safety risks and propose corresponding mitigation strategies. Furthermore, LLM-based chatbot development in oncology should explicitly document prompt design and system configuration in accordance with validated reporting guidelines.

## Supplementary material

10.2196/96241 Multimedia Appendix 1 Search string.

10.2196/96241 Multimedia Appendix 2 Full-text screening decision at full-text screening.

10.2196/96241 Multimedia Appendix 3 Reasons for exclusion during title or abstract screening (counts not mutually exclusive).

10.2196/96241 Checklist 1 PRISMA checklist.

## Abbreviations

CHART: Chatbot Assessment Reporting Tool
JBI: Joanna Briggs Institute
LLM: large language model
NLP: natural language processing
OSF: Open Science Framework
PRISMA: Preferred Reporting Items for Systematic Reviews and Meta-Analyses
PRISMA-ScR: Preferred Reporting Items for Systematic Reviews and Meta-Analyses extension for Scoping Reviews
PRO: patient-reported outcome
RAG: retrieval-augmented generation
TRIPOD-LLM: Transparent Reporting of a Multivariable Prediction Model for Individual Prognosis or Diagnosis–Large Language Models
