# Supplementary material for: Multiturn Large Language Model–Based Conversational Agents for Patients With Cancer and Caregivers: Scoping Review
Source: JMIR Cancer. 2026 Jul 21;12:e96241. doi: 10.2196/96241 (PMC13387488; doi:10.2196/96241)
Supplement: Multimedia Appendix 1 [file cancer-v12-e96241-s001.docx]

**Supplement 1. Search String**

| **Database** | **Number of articles retrieved** |
| --- | --- |
| **PubMed** | **163** |
| 1. ("Neoplasms"[Mesh] OR cancer*[Title/Abstract] OR neoplasm*[Title/Abstract] OR carcinoma*[Title/Abstract] OR oncol*[Title/Abstract] OR malignan*[Title/Abstract] OR tumor*[Title/Abstract] OR leukemia*[Title/Abstract] OR leukaemia*[Title/Abstract] OR sarcoma*[Title/Abstract] OR lymphoma*[Title/Abstract] OR melanoma*[Title/Abstract] OR blastoma*[Title/Abstract] OR myeloma*[Title/Abstract]) 2. "chatbot*"[Title/Abstract] OR "chat bot*"[Title/Abstract] OR "digital assistant*"[Title/Abstract] OR "digital agent*"[Title/Abstract] OR "ai bot*"[Title/Abstract] OR "virtual coach*"[Title/Abstract] OR "artificial agent*"[Title/Abstract] OR "virtual agent*"[Title/Abstract] OR "virtual assistant*"[Title/Abstract] OR "assistance technolog*"[Title/Abstract] OR "social robot*"[Title/Abstract] OR "conversational AI"[Title/Abstract] OR "nurse avatar*"[Title/Abstract] OR "nursing avatar*"[Title/Abstract] OR "virtual avatar*"[Title/Abstract] OR "chatterbot*"[Title/Abstract] OR "social bot*"[Title/Abstract] OR "chat robot*"[Title/Abstract] OR "relational agent*"[Title/Abstract] OR "dialogue system*"[Title/Abstract] OR "dialog system*"[Title/Abstract] OR "conversational system*"[Title/Abstract] OR "conversational agent*"[Title/Abstract] OR "question answer* system*"[Title/Abstract] OR "intelligent agent*"[Title/Abstract] 3. "Large Language Models"[MeSH Terms] OR "Generative Artificial Intelligence"[MeSH Terms] OR "large language model*"[Title/Abstract] OR "llm"[Title/Abstract] OR "generative AI"[Title/Abstract] OR "Generative Artificial Intelligence"[Title/Abstract] OR "foundation model*"[Title/Abstract] OR "ChatGPT"[Title/Abstract] OR "GPT-4"[Title/Abstract] OR "GPT-3.5"[Title/Abstract] OR "Claude"[Title/Abstract] OR "Gemini"[Title/Abstract] OR "Bard"[Title/Abstract] OR "Llama"[Title/Abstract] OR "Mistral"[Title/Abstract] OR "Phi-3"[Title/Abstract] OR "PaLM"[Title/Abstract] OR "fine tuned model*"[Title/Abstract] 4. "Patients"[MeSH Terms] OR "Caregivers"[MeSH Terms] OR "Family"[MeSH Terms] OR "patient*"[Title/Abstract] OR "individual*"[Title/Abstract] OR "person*"[Title/Abstract] OR "caregiver*"[Title/Abstract] OR "carer*"[Title/Abstract] OR "care giver*"[Title/Abstract] OR "family member*"[Title/Abstract] OR "informal care*"[Title/Abstract] OR "unpaid care*"[Title/Abstract] OR "spouse*"[Title/Abstract] OR "husband*"[Title/Abstract] OR "wife*"[Title/Abstract] OR "partner*"[Title/Abstract] OR "parent*"[Title/Abstract] OR "daughter*"[Title/Abstract] OR "son"[Title/Abstract] OR "sibling*"[Title/Abstract] OR "support person*"[Title/Abstract] OR "home care*"[Title/Abstract] OR "guardian*"[Title/Abstract] 5. #1 AND #2 AND #3 AND #4 |  |
| **Embase** | **1,063** |
| 1. 'neoplasm'/exp OR cancer*:ab,ti OR neoplasm*:ab,ti OR carcinoma*:ab,ti OR oncol*:ab,ti OR malignan*:ab,ti OR tumor*:ab,ti OR tumour*:ab,ti OR leukemia*:ab,ti OR leukaemia*:ab,ti OR sarcoma*:ab,ti OR lymphoma*:ab,ti OR melanoma*:ab,ti OR blastoma*:ab,ti OR myeloma*:ab,ti 2. 'chatbot'/exp OR 'conversational agent'/exp OR chatbot*:ab,ti OR 'chat bot*':ab,ti OR 'digital assistant*':ab,ti OR 'digital agent*':ab,ti OR 'ai bot*':ab,ti OR 'virtual coach*':ab,ti OR 'artificial agent*':ab,ti OR 'virtual agent*':ab,ti OR 'virtual assistant*':ab,ti OR 'assistance technolog*':ab,ti OR 'social robot*':ab,ti OR 'conversational AI':ab,ti OR 'nurse avatar*':ab,ti OR 'nursing avatar*':ab,ti OR 'virtual avatar*':ab,ti OR chatterbot*:ab,ti OR 'social bot*':ab,ti OR 'chat robot*':ab,ti OR 'relational agent*':ab,ti OR 'dialogue system*':ab,ti OR 'dialog system*':ab,ti OR 'conversational system*':ab,ti OR 'conversational agent*':ab,ti OR 'question answer* system*':ab,ti OR 'intelligent agent*':ab,ti 3. 'large language model'/exp OR 'generative artificial intelligence'/exp OR 'large language model*':ab,ti OR LLM*:ab,ti OR 'generative AI':ab,ti OR 'generative artificial intelligence':ab,ti OR 'foundation model*':ab,ti OR ChatGPT:ab,ti OR 'GPT-4':ab,ti OR 'GPT-3.5':ab,ti OR Claude:ab,ti OR Gemini:ab,ti OR Bard:ab,ti OR Llama:ab,ti OR Mistral:ab,ti OR 'Phi-3':ab,ti OR PaLM:ab,ti OR 'instruction-tuned model*':ab,ti OR 'fine-tuned model*':ab,ti 4. 'patient'/exp OR 'caregiver'/exp OR 'family'/exp OR patient*:ab,ti OR individual*:ab,ti OR person*:ab,ti OR caregiver*:ab,ti OR carer*:ab,ti OR 'care giver*':ab,ti OR 'family member*':ab,ti OR 'informal care*':ab,ti OR 'unpaid care*':ab,ti OR spouse*:ab,ti OR husband*:ab,ti OR wife*:ab,ti OR partner*:ab,ti OR parent*:ab,ti OR daughter*:ab,ti OR son*:ab,ti OR sibling*:ab,ti OR 'support person*':ab,ti OR 'home care*':ab,ti OR guardian*:ab,ti 5. #1 AND #2 AND #3 AND #4 |  |
| **Scopus** | **289** |
| 1. TITLE-ABS-KEY ( cancer* OR neoplasm* OR carcinoma* OR oncol* OR malignan* OR tumor* OR tumour* OR leukemia* OR leukaemia* OR sarcoma* OR lymphoma* OR melanoma* OR blastoma* OR myeloma* ) 2. TITLE-ABS-KEY ( chatbot* OR "chat bot*" OR "digital assistant*" OR "digital agent*" OR "ai bot*" OR "virtual coach*" OR "artificial agent*" OR "virtual agent*" OR "virtual assistant*" OR "assistance technolog*" OR "social robot*" OR "conversational AI" OR "nurse avatar*" OR "nursing avatar*" OR "virtual avatar*" OR chatterbot* OR "social bot*" OR "chat robot*" OR "relational agent*" OR "dialogue system*" OR "dialog system*" OR "conversational system*" OR "conversational agent*" OR "question answer* system*" OR "intelligent agent*" ) 3. TITLE-ABS-KEY ( "large language model*" OR llm* OR "generative AI" OR "generative artificial intelligence" OR "foundation model*" OR chatgpt OR "GPT-4" OR "GPT-3.5" OR claude OR gemini OR bard OR llama OR mistral OR "Phi-3" OR palm OR "instruction-tuned model*" OR "fine-tuned model*" ) 4. TITLE-ABS-KEY ( patient* OR individual* OR person* OR caregiver* OR carer* OR "care giver*" OR "family member*" OR "informal care*" OR "unpaid care*" OR spouse* OR husband* OR wife* OR partner* OR parent* OR daughter* OR son* OR sibling* OR "support person*" OR "home care*" OR guardian* ) 5. #1 AND #2 AND #3 AND #4 |  |
| **Web of Science** | **157** |
| 1. TS=(cancer* OR neoplasm* OR carcinoma* OR oncol* OR malignan* OR tumor* OR tumour* OR leukemia* OR leukaemia* OR sarcoma* OR lymphoma* OR melanoma* OR blastoma* OR myeloma*) 2. TS=(chatbot* OR "chat bot*" OR "digital assistant*" OR "digital agent*" OR "ai bot*" OR "virtual coach*" OR "artificial agent*" OR "virtual agent*" OR "virtual assistant*" OR "assistance technolog*" OR "social robot*" OR "conversational AI" OR "nurse avatar*" OR "nursing avatar*" OR "virtual avatar*" OR "chatterbot*" OR "social bot*" OR "chat robot*" OR "relational agent*" OR "dialogue system*" OR "dialog system*" OR "conversational system*" OR "conversational agent*" OR "question answer* system*" OR "intelligent agent*") 3. TS=("large language model*" OR LLM* OR "generative AI" OR "generative artificial intelligence" OR "foundation model*" OR ChatGPT OR "GPT-4" OR "GPT-3.5" OR Claude OR Gemini OR Bard OR Llama OR Mistral OR "Phi-3" OR PaLM OR "instruction-tuned model*" OR "fine-tuned model*") 4. TS=(patient* OR individual* OR person* OR caregiver* OR carer* OR "care giver*" OR "family member*" OR "informal care*" OR "unpaid care*" OR spouse* OR husband* OR wife* OR partner* OR parent* OR daughter* OR son* OR sibling* OR "support person*" OR "home care*" OR guardian*) 5. #1 AND #2 AND #3 AND #4 |  |
| **CINAHL** | **60** |
| 1. (MH "Neoplasms+") OR XB (cancer* OR neoplasm* OR carcinoma* OR oncol* OR malignan* OR tumor* OR tumour* OR leukemia* OR leukaemia* OR sarcoma* OR lymphoma* OR melanoma* OR blastoma* OR myeloma*) 2. (MH "Chatbots") OR (MH "Artificial Intelligence") OR XB (chatbot* OR "chat bot*" OR "digital assistant*" OR "digital agent*" OR "ai bot*" OR "virtual coach*" OR "artificial agent*" OR "virtual agent*" OR "virtual assistant*" OR "assistance technolog*" OR "social robot*" OR "conversational AI" OR "nurse avatar*" OR "nursing avatar*" OR "virtual avatar*" OR "chatterbot*" OR "social bot*" OR "chat robot*" OR "relational agent*" OR "dialogue system*" OR "dialog system*" OR "conversational system*" OR "conversational agent*" OR "question answer* system*" OR "intelligent agent*") 3. (MH "Generative Artificial Intelligence") OR XB ("large language model*" OR LLM* OR "generative AI" OR "generative artificial intelligence" OR "foundation model*" OR ChatGPT OR "GPT-4" OR "GPT-3.5" OR Claude OR Gemini OR Bard OR Llama OR Mistral OR "Phi-3" OR PaLM OR "instruction-tuned model*" OR "fine-tuned model*") 4. (MH "Patients+" OR MH "Caregivers" OR MH "Family") OR XB (patient* OR caregiver* OR carer* OR "care giver*" OR "family member*" OR "informal care*" OR "unpaid care*" OR spouse* OR husband* OR wife* OR partner* OR parent* OR daughter* OR son* OR sibling* OR "support person*" OR "home care*" OR guardian*) 5. #1 AND #2 AND #3 AND #4 |  |
| **PsycINFO** | **9** |
| 1. (DE "Neoplasms") OR XB("cancer*" OR "neoplasm*" OR "carcinoma*" OR "oncol*" OR "malignan*" OR "tumor*" OR "tumour*" OR "leukemia*" OR "leukaemia*" OR "sarcoma*" OR "lymphoma*" OR "melanoma*" OR "blastoma*" OR "myeloma*") 2. (DE "Artificial Intelligence" OR DE "Human Computer Interaction") OR XB (chatbot* OR "chat bot*" OR "digital assistant*" OR "digital agent*" OR "ai bot*" OR "virtual coach*" OR "artificial agent*" OR "virtual agent*" OR "virtual assistant*" OR "assistance technolog*" OR "social robot*" OR "conversational AI" OR "nurse avatar*" OR "nursing avatar*" OR "virtual avatar*" OR "chatterbot*" OR "social bot*" OR "chat robot*" OR "relational agent*" OR "dialogue system*" OR "dialog system*" OR "conversational system*" OR "conversational agent*" OR "question answer* system*" OR "intelligent agent*") 3. XB ("large language model*" OR LLM* OR "generative AI" OR "generative artificial intelligence" OR "foundation model*" OR ChatGPT OR "GPT-4" OR "GPT-3.5" OR Claude OR Gemini OR Bard OR Llama OR Mistral OR "Phi-3" OR PaLM OR "instruction-tuned model*" OR "fine-tuned model*") 4. (DE "Patients+" OR DE "Caregivers" OR DE "Family") OR XB (patient* OR caregiver* OR carer* OR "care giver*" OR "family member*" OR "informal care*" OR "unpaid care*" OR spouse* OR husband* OR wife* OR partner* OR parent* OR daughter* OR son* OR sibling* OR "support person*" OR "home care*" OR guardian*) 5. #1 AND #2 AND #3 AND #4 |  |
| **IEEE Xplore Digital Library** | **10** |
| 1. ("Abstract":cancer* OR "Abstract":neoplasm OR "Abstract":carcinoma OR "Abstract":oncology OR "Abstract":malignant OR "Abstract":tumor OR "Abstract":tumour OR "Abstract":leukemia OR "Abstract":sarcoma OR "Abstract":lymphoma OR "Abstract":melanoma OR "Abstract":myeloma) 2. ("Abstract":chatbot* OR "Abstract":"chat bot" OR "Abstract":"digital assistant" OR "Abstract":"digital agent" OR "Abstract":"virtual agent" OR "Abstract":"virtual assistant" OR "Abstract":"conversational AI" OR "Abstract":chatterbot OR "Abstract":"relational agent" OR "Abstract":"dialogue system" OR "Abstract":"dialog system" OR "Abstract":"conversational agent" OR "Abstract":"intelligent agent") 3. ("Abstract":"large language model" OR "Abstract":LLM OR "Abstract":"generative AI" OR "Abstract":ChatGPT OR "Abstract":"GPT-4" OR "Abstract":"GPT-3.5" OR "Abstract":Claude OR "Abstract":Gemini OR "Abstract":Llama OR "Abstract":Mistral OR "Abstract":"foundation model") 4. ("Abstract":patient* OR "Abstract":caregiver* OR "Abstract":carer OR "Abstract":"family member" OR "Abstract":"informal care" OR "Abstract":spouse OR "Abstract":parent* OR "Abstract":guardian OR "Abstract":"home care")   #1 AND #2 AND #3 AND #4 |  |
| **ACM Digital Library.** | **1** |
| 1. (Abstract:cancer* OR Abstract:neoplasm* OR Abstract:carcinoma* OR Abstract:oncol* OR Abstract:malignan* OR Abstract:tumor* OR Abstract:tumour* OR Abstract:leukemia* OR Abstract:leukaemia* OR Abstract:sarcoma* OR Abstract:lymphoma* OR Abstract:melanoma* OR Abstract:blastoma* OR Abstract:myeloma* OR Title:cancer* OR Title:neoplasm* OR Title:carcinoma* OR Title:oncol* OR Title:malignan* OR Title:tumor* OR Title:tumour* OR Title:leukemia* OR Title:leukaemia* OR Title:sarcoma* OR Title:lymphoma* OR Title:melanoma* OR Title:blastoma* OR Title:myeloma*) 2. (Abstract:"chatbot*" OR Abstract:"chat bot*" OR Abstract:"conversational agent*" OR Abstract:"conversational AI" OR Abstract:"virtual assistant*" OR Abstract:"digital assistant*" OR Abstract:"virtual agent*" OR Abstract:"digital agent*" OR Abstract:"dialogue system*" OR Abstract:"dialog system*" OR Abstract:"conversational system*" OR Abstract:"relational agent*" OR Abstract:"intelligent agent*" OR Abstract:"social robot*" OR Abstract:"chatterbot*" OR Title:"chatbot*" OR Title:"chat bot*" OR Title:"conversational agent*" OR Title:"conversational AI") 3. (Abstract:"large language model*" OR Abstract:LLM OR Abstract:"generative AI" OR Abstract:"generative artificial intelligence" OR Abstract:"foundation model*" OR Abstract:ChatGPT OR Abstract:"GPT-4" OR Abstract:"GPT-3.5" OR Abstract:Claude OR Abstract:Gemini OR Abstract:Llama OR Abstract:Mistral OR Abstract:"Phi-3" OR Abstract:PaLM OR Abstract:"transformer-based model*" OR Abstract:"generative pretrained transformer*" OR Abstract:"instruction-tuned model*" OR Abstract:"fine-tuned model*" OR Title:"large language model*" OR Title:ChatGPT OR Title:LLM OR Title:"generative AI") 4. (Abstract:patient* OR Abstract:caregiver* OR Abstract:carer* OR Abstract:"care giver*" OR Abstract:"family member*" OR Abstract:"informal care*" OR Abstract:spouse* OR Abstract:parent* OR Abstract:"home care*" OR Title:patient* OR Title:caregiver* OR Title:carer*)   #1 AND #2 AND #3 AND #4 |  |

**Supplement 2 Full-text Screening Decision at Full-text Screening**

| Study | 1^st^ Decision | | Reason | 2^nd^ Decision | | Reason |
| --- | --- | --- | --- | --- | --- | --- |
| Chen et al., 2024 | YRJ | Include | The study explicitly implemented both short-term and long-term dialogue history management. |  | |  |
|  | HJC | Include |  |  |  |  |
| Lee et al., 2024 | YRJ | Borderline | Although the study recorded conversation history, whether responses were generated based on prior conversational context is unclear. | YRJ | Include | Given that the architecture suggests responses were likely generated based on conversational history, the study was included. |
|  | HJC | Include | The model developed in this study is likely to generate medical responses based on conversational history. | HJC | Include |  |
| Akdogan et al., 2025 | YRJ | Borderline | Whether responses were generated based on prior conversational context was not explicitly described. | YRJ | Include | The introduction explicitly references ChatGPT's conversational functionality as a key rationale for the study. Given that ChatGPT-4.0 inherently supports conversation history management, the study was considered likely to involve multi-turn interaction and included through reviewer consensus. |
|  | HJC | Exclude | The study provided limited explicit reporting of multi-turn conversational functionality. | HJC | Include |  |
| Bharambe et al, 2025 | YRJ | Include | The study proposed a chatbot architecture designed to facilitate ongoing patient-chatbot interaction. |  | |  |
|  | HJC | Include |  |  |  |  |
| Boie et al., 2025 | YRJ | Include | The system prompt explicitly instructed the chatbot to ask clarifying questions when necessary, indicating intentional design for ongoing conversational interaction. |  | |  |
|  | HJC | Include |  |  |  |  |
| Hasei et al., 2025 | YRJ | Include | The study explicitly implemented history-based memory retention and contextually appropriate follow-up questioning. |  | |  |
|  | HJC | Include |  |  |  |  |
| Mclnerney et al., 2025 | YRJ | Include | The study developed a patient-facing chatbot with the explicit goal of providing personalized, interactive support, including follow-up questioning functionality. |  | |  |
|  | HJC | Include |  |  |  |  |
| Sugan et al., 2025 | YRJ | Borderline | The study developed a conversational AI prototype designed to support ongoing caregiver interaction, but explicit reporting of conversational functionality was limited. | YRJ | Include | The study evaluated a functional LLM-based conversational AI prototype with real caregiver engagement, supporting its use in ongoing conversational interaction. An inclusive approach was adopted given the nascent state of this research field. |
|  | HJC | Exclude | The study provided limited explicit reporting of multi-turn conversational functionality. | HJC | Include |  |

**Supplement 3. Reasons for exclusion during title/abstract screening (counts not mutually exclusive)**

| **Reason for exclusion** | **Frequency (n)** |
| --- | --- |
| Did not primarily target patients or caregivers | 499 |
| Not a conversational chatbot | 453 |
| Not relevant to the research objective | 448 |
| Did not address a cancer-related context | 113 |
| Publication type did not meet inclusion criteria | 72 |
| Did not use a large language model (LLM) | 54 |
| Not published in English | 5 |
| Not conducted in an adult population | 1 |
