# Supplementary material for: Multiturn Large Language Model–Based Conversational Agents for Patients With Cancer and Caregivers: Scoping Review
Source: JMIR Cancer. 2026 Jul 21;12:e96241. doi: 10.2196/96241 (PMC13387488; doi:10.2196/96241)
Supplement: Multimedia Appendix 2 [file cancer-v12-e96241-s002.docx]

**Supplement 2 Full-text Screening Decision at Full-text Screening**

| Study | 1^st^ Decision | | Reason | 2^nd^ Decision | | Reason |
| --- | --- | --- | --- | --- | --- | --- |
| Chen et al., 2024 | YRJ | Include | The study explicitly implemented both short-term and long-term dialogue history management. |  | |  |
|  | HJC | Include |  |  |  |  |
| Lee et al., 2024 | YRJ | Borderline | Although the study recorded conversation history, whether responses were generated based on prior conversational context is unclear. | YRJ | Include | Given that the architecture suggests responses were likely generated based on conversational history, the study was included. |
|  | HJC | Include | The model developed in this study is likely to generate medical responses based on conversational history. | HJC | Include |  |
| Akdogan et al., 2025 | YRJ | Borderline | Whether responses were generated based on prior conversational context was not explicitly described. | YRJ | Include | The introduction explicitly references ChatGPT's conversational functionality as a key rationale for the study. Given that ChatGPT-4.0 inherently supports conversation history management, the study was considered likely to involve multi-turn interaction and included through reviewer consensus. |
|  | HJC | Exclude | The study provided limited explicit reporting of multi-turn conversational functionality. | HJC | Include |  |
| Bharambe et al, 2025 | YRJ | Include | The study proposed a chatbot architecture designed to facilitate ongoing patient-chatbot interaction. |  | |  |
|  | HJC | Include |  |  |  |  |
| Boie et al., 2025 | YRJ | Include | The system prompt explicitly instructed the chatbot to ask clarifying questions when necessary, indicating intentional design for ongoing conversational interaction. |  | |  |
|  | HJC | Include |  |  |  |  |
| Hasei et al., 2025 | YRJ | Include | The study explicitly implemented history-based memory retention and contextually appropriate follow-up questioning. |  | |  |
|  | HJC | Include |  |  |  |  |
| Mclnerney et al., 2025 | YRJ | Include | The study developed a patient-facing chatbot with the explicit goal of providing personalized, interactive support, including follow-up questioning functionality. |  | |  |
|  | HJC | Include |  |  |  |  |
| Sugan et al., 2025 | YRJ | Borderline | The study developed a conversational AI prototype designed to support ongoing caregiver interaction, but explicit reporting of conversational functionality was limited. | YRJ | Include | The study evaluated a functional LLM-based conversational AI prototype with real caregiver engagement, supporting its use in ongoing conversational interaction. An inclusive approach was adopted given the nascent state of this research field. |
|  | HJC | Exclude | The study provided limited explicit reporting of multi-turn conversational functionality. | HJC | Include |  |
