# Supplementary material for: Multiturn Large Language Model–Based Conversational Agents for Patients With Cancer and Caregivers: Scoping Review
Source: JMIR Cancer. 2026 Jul 21;12:e96241. doi: 10.2196/96241 (PMC13387488; doi:10.2196/96241)
Supplement: Multimedia Appendix 3 [file cancer-v12-e96241-s003.docx]

**Supplement 3. Reasons for exclusion during title/abstract screening (counts not mutually exclusive)**

| **Reason for exclusion** | **Frequency (n)** |
| --- | --- |
| Did not primarily target patients or caregivers | 499 |
| Not a conversational chatbot | 453 |
| Not relevant to the research objective | 448 |
| Did not address a cancer-related context | 113 |
| Publication type did not meet inclusion criteria | 72 |
| Did not use a large language model (LLM) | 54 |
| Not published in English | 5 |
| Not conducted in an adult population | 1 |
